# HIF1α isoforms in benign and malignant prostate tissue and their correlation to neuroendocrine differentiation

**DOI:** 10.1186/1471-2407-10-385

**Published:** 2010-07-21

**Authors:** Nastaran Monsef, Maria Soller, Ioannis Panagopoulos, Per Anders Abrahamsson

**Affiliations:** 1 Department of Laboratory Medicine, Division of Pathology, Lund University Hospital, Sweden; 2 Department of Clinical Genetics, Lund University Hospital, Sweden; 3 Department of Clinical Sciences, Division of Urological Cancer, Malmö University Hospital, Lund University, Sweden

## Abstract

**Background:**

Neuroendocrine (NE) differentiation in prostate cancer has been correlated with a poor prognosis and hormone refractory disease. In a previous report, we demonstrated the presence of immunoreactive cytoplasmic hypoxia inducible factor 1α (HIF1α), in both benign and malignant NE prostate cells. HIF1α and HIF1β are two subunits of HIF1, a transcription factor important for angiogenesis. The aim of this study was to elucidate whether the cytoplasmic stabilization of HIF1α in androgen independent NE differentiated prostate cancer is due to the presence of certain HIF1α isoforms.

**Methods:**

We studied the HIF1α isoforms present in 8 cases of benign prostate hyperplasia (BPH) and 43 cases of prostate cancer with and without NE differentiation using RT-PCR, sequencing analysis, immunohistochemistry and *in situ *hybridization.

**Results:**

We identified multiple isoforms in both benign and malignant prostate tissues. One of these isoforms, *HIF1α1.2*, which was previously reported to be testis specific, was found in 86% of NE-differentiated prostate tumors, 92% of HIF1α immunoreactive prostate tumors and 100% of cases of benign prostate hyperplasia. Immunohistochemistry and *in situ *hybridization results showed that this isoform corresponds to the cytoplasmic HIF1α present in androgen-independent NE cells of benign and malignant prostate tissue and co-localizes with immunoreactive cytoplasmic HIF1β.

**Conclusion:**

Our results indicate that the cytoplasmic stabilization of HIF1α in NE-differentiated cells in benign and malignant prostate tissue is due to presence of an HIF1α isoform, HIF1α1.2. Co-localization of this isoform with HIF1β indicates that the HIF1α1.2 isoform might sequester HIF1β in the cytoplasm.

## Background

Neuroendocrine (NE) cells in benign and malignant prostate tissue are characterized by expression of the NE marker chromogranin A and their specific morphology, including cytoplasmic extensions and dendritic processes. In addition, they lack androgen receptor and are therefore androgen-independent [1,2]. The majority of prostatic adenocarcinomas contain tumor cells with NE features which are present focally throughout the tumors. Several studies have shown that an increased number of NE cells in prostate cancer tissue and increased levels of circulating chromogranin A, an NE marker usually expressed in NE cells, are associated with a poor prognosis, tumor progression and androgen-independent disease [1,2]. It has been hypothesized that NE cells might affect the growth and proliferation of prostate tumors in an androgen receptor-independent way, probably through the secretion of NE peptides and autocrine/paracrine signaling pathways [1,3-6]. NE tumor cells in prostate cancer may also have a role in tumor angiogenesis. Increased expression of vascular endothelial growth factor (VEGF) in malignant NE cells and a higher density of microvessels around nests of such cells compared to areas of prostate cancer without these cells have been reported [7]. In a previous study, we demonstrated the presence of cytoplasmic hypoxia inducible factor 1α (HIF1α) in a population of NE cells in benign and malignant prostate tissues [8]. HIFs are a family of transcription factors that are essential for angiogenesis and the adaptation of cells to hypoxia in both physiological and pathological processes [9,10]. The possible mechanisms behind HIF1α stabilization and the function of cytoplasmic HIF1α in NE cells of benign and malignant prostate tissues are not known. However, the complicated regulation of HIFs has been extensively studied in recent years. HIF1, which is composed of two subunits, HIF1α and β, binds to the HRE of promoter regions and activates many genes involved in angiogenesis, glycolysis and anaerobic metabolism. In normoxia, the HIF1α subunit is hydroxylated on specific proline residues (402 and 564) within the oxygen dependent domain (ODD) by specific oxygen-sensitive prolyl hydroxylases and, then ubiquitinated through the von Hippel-Lindau (VHL) dependent pathway and degraded instantly in the proteasome [9,10]. However, in hypoxic conditions, HIF1α is stabilized by impaired function of these prolyl hydroxylases. After its interaction with a series of small proteins, such as P300 and CBP, the HIF1α and β complex translocates to the nucleus using its nuclear localizing signal (NLS) domains at C-and N-terminus and activates the cell's adaptive response to hypoxia [9,10]

There have been several previous reports on HIF1α isoforms lacking several exons or displaying different exons than the wild type protein. Some of these isoforms encode cytoplasmic HIF1α protein or proteins with altered transcriptional activity compared to the wild type protein. Regarding the N-terminal domain, two HIF1α isoforms with different first exons have been identified: HIF1α1.2, a protein with a different first and second exon which is 59 amino acids shorter than wild type HIF1α and encodes a cytoplasmic protein specifically expressed in the human testis [11]; HIF1α1.3, which is present in activated T-lymphocytes and encodes a functional protein with weaker transcriptional activity that the wild type protein [12]. At the C-terminal domain, isoforms lacking either exon 12 [13] or exons 11 and 12 [14] have been reported. Both isoforms have been shown to be stable cytoplasmic proteins and inhibit the function of full-length HIF1α. Another shorter HIF1α isoform lacking exon 14 has also been reported [15]. This isoform was shown to be 3-fold less active than full-length HIF1α.

The aim of this study was to elucidate the mechanism behind the cytoplasmic stabilization of HIF1α in NE cells of benign and malignant prostate tissues. We have investigated whether the cytoplasmic HIF1α corresponds to any specific known isoform. Based on the sequences published by the National Center for Biotechnology Information (NCBI), we designed primers to amplify known HIF1α isoforms and determined their presence in benign prostate hyperplasia and prostate cancer with and without NE differentiation.

## Methods

### Study cases

The present study included tissue obtained from 51 patients undergoing transurethral resection of the prostate (TURP): From 8 patients with obstructive voiding symptoms due to benign hyperplasia of the prostate (BPH), and from 43 patients with prostate cancer. The majority of the patients in the latter group had previously received therapy and displayed hormone-refractory disease (Table 1). All tissue specimens were examined by a National Board certified pathologist (NM). This study was approved by the Ethics Committee of Lund University, and the Helsinki Declaration regarding the use of human tissue was followed.

**Table 1:** Characteristics of patients whose samples were used in this study

	Nr. of cases
Age	
<65	7
≥65	44
Benign prostate hyperplasia	8
Adenocarcinoma	43
Gleason Score	
5	1
6	2
7	11
8	3
9	13
10	3
Non - determined	10
Clinical stage	
T1	3
T2	12
T3	19
T4	2
Non - determined	7
Hormone refractory adenocarcinoma	30
Adenocarcinoma with non determined hormonal state	13

### RNA preparation, RT-PCR and sequence analysis

Total RNA was extracted from prostate tissue using TRIzol reagent (Invitrogen, Carlsbad, CA), and cDNA synthesis was conducted as described previously. To detect isoforms of HIF1α, PCR was performed with the primer sets shown in Table 2. The 50-μl reaction volume contained 20 mM Tris-HCl (pH 8.4), 50 mM KCl, 1.5 mM MgCl_2_, 0.2 mM of each dNTP, 1 unit Platinum Taq DNA polymerase (Invitrogen), 10 μM of each of the forward and reverse primers and 1 μl of cDNA. After an initial denaturation at 94°C for 5 min, 40 cycles of 1 min at 94°C, 1 min at 56°C and 1 min at 72°C were run using a Master cycler gradient (Eppendorf-Netheler. Hinz GmbH, Hamburg, Germany) termam cycler. The size of the amplified PCR product was determined on an agarose gel with 1 kb DNA ladder (cat. no. 15615-024, Invitrogen) and. For sequence analysis, the amplified fragments were purified using the Qiagen PCR purification kit (Qiagen), sequenced using the dideoxy procedure with an ABI Prism Big Dye terminator v1.1 cycle sequencing kit (PE Applied Biosystems) and the same primers as for PCR on an Applied Biosystems Model 3100-Avant Genetic Analyzer.

**Table 2:** Primers used for amplification/sequencing and biotinylated oligonucleotide probes

	Forward primer 5'-3'	Reverse primer 5'-3'	Size of amplified fragment lengths (bp)
HIF1α 1.1	CACCTCTGGAC TTGCCTTTCCTTC	CACCAGCATCCAG AAGTTTCCTCAC	351
aHIF1α 1.2	ATGTGCAAGAC ACTGCATTCTTAG	CACCAGCATCCAG AAGTTTCCTCAC	220
HIF1α 1.3	TGGTGGTTACTC AGCACTTTTAGA	CACCAGCATCCAG AAGTTTCCTCAC	-
HIF1α 10-13	GGCAATGTCTCC ATTACCCACCG	CGCTTTCTCTGAGC ATTCTGCAAG	791
HIF1α12	GGTGGCATTACG AGTAGGTTCTTGT	AGGTCCCTATATC CCAATGGATGA	180
HIF1α 13-15	ACTCAAAGTCGG ACAGCCTCACCA	ATCCATTGATTGC CCCAGCAGTC	381
Probe HIF1α 1.2	Antisense, Biotin-GC AAGATATGTG CAAGACAC-Biotin	Sense, Biotin-GTGT CTTGCACAT ATCTTGC-Biotin	-

### *In situ *hybridization

Biotinylated anti sense and sense olignucleotide probes were designed and purchased from Invitrogen (Table 2). Tissues were fixed in 4% formaldehyde, 4-μm-thick sections were cut and mounted on silanized slides (DAKO S3003), dewaxed with xylene for 10 min and rehydrated stepwise with 100% ethanol for 3 min and 70% ethanol for 3 min. Endogenous peroxidase and biotin were blocked with peroxidase blocking solution (DAKO REAL, S2023) and biotin blocking system (DAKO, X0590) for 15 min each. The sections were then permeabilized with Proteinase K (DAKO S3020) and washed with Tris-buffered saline three times. Biotinylated probes designed for HIF1α1.2 (Table 2, Invitrogen) were mixed in hybridization buffer (50% formamide) at a concentration of 200 ng/ml. Probes were added to the sections, which were then covered with coverslips and heated at 92°C for 5 min. Hybridization was carried out in a humid chamber at 37°C overnight. The sections were then washed in 2 × SSC for 10 min followed by three washes in Tris-buffered saline. For detection of hybridization signals, the Gen Point Tyramid Signal Amplification System (Dako, K0620) with chromogenic substrate (3.3'diaminobenzidine) was used according to the manufacturer's instructions.

### Immunohistochemistry

Tissue samples were fixed in 4% buffered formalin and embedded in paraffin. One appropriate block from each specimen was selected and serial 4-μm-thick sections of tissue measuring at least 0,5 × 0,5 cm was cut from the paraffin lock. The sections were used for immunohistochemical analysis and histological examination after hematoxylin and eosin staining. After deparaffinization and rehydration, the sections were heated in citrate buffer solution (pH 6) for 14 min in a microwave oven for antigen retrieval. The characteristics and working concentrations of the antibodies used in this study are summarized in Table 3. Three primary HIF1α antibodies with different epitopes were chosen; polyclonal antibody (AB1, Y-15, santa cruz) recognizing an epitope near the N-terminus of wild type HIF1α, monoclonal HIF1α (Ab2, Novus Biological, NB 100-123), which recognizes an epitope in the middle of wild type HIF1α (residues 432-528, corresponding to exon 10 and 11 of HIF1α protein) and polyclonal HIF1α (Ab3, Bethyl Laboratories), which recognizes a region between residues 775 and the C-terminus (residue 826), corresponding to the exon 14 of HIF1α protein. Chromogranin A was chosen as NE markers, and a polyclonal anti-chromogranin A antibody (DAKO, Denmark) was used. Immunohistochemical staining was carried out with LSAB+ System (HRP, K0670, DAKO, Denmark), according to the manufacturer's instructions. Sections were incubated with primary antibody at room temperature for 60 min. Counterstaining was done with Mayer hematoxylin. Tumors with chromogranin A immunopositivity were considered NE-differentiated. The smallest chromogranin A immunopositive tumor area, detected in our study cases, contained 10 immunostained cells.

**Table 3:** Antibodies used for immunostaining

Antibodies	Clone/Code	Dilution	Source
HIF1α, Ab1	Goat polyclonal Y-15	1:50	Santa Cruz
HIF1α, Ab2	Mouse monoclonal, NB 100-123	1:1000	Novus biological
HIF1α, Ab3	Rabbit polyclonal	1:500	Bethyl laboratories
HIF1β	Mouse monoclonal, ab465-100	1:100	Abcam
Androgen receptor, AR	Mouse monoclonal AR441	1:1000	DAKO
Chromogranin A	Rabbit polyclonal IHC00460	1:1000	DAKO

Double immunostaining was performed with combinations of primary antibodies for androgen receptor/chromogranin A and HIF1β/HIF1αA3. Double immunostaining kit (PicTure™ Invitrogen, catalog nr. 87-9999) was used, according to the manufacturer's instructions. Goat anti mouse IgG HRP (horseradish peroxidase) conjugate used with DAB (3,3' Diaminobenzidine) produced a brown stain. Goat anti Rabbit IgG-AP polymer conjugate used with Fast red produced a red stain.

## Results

### A broad range of *HIF1α *isoforms exists in benign and malignant prostate tissues

**Figure 1:**
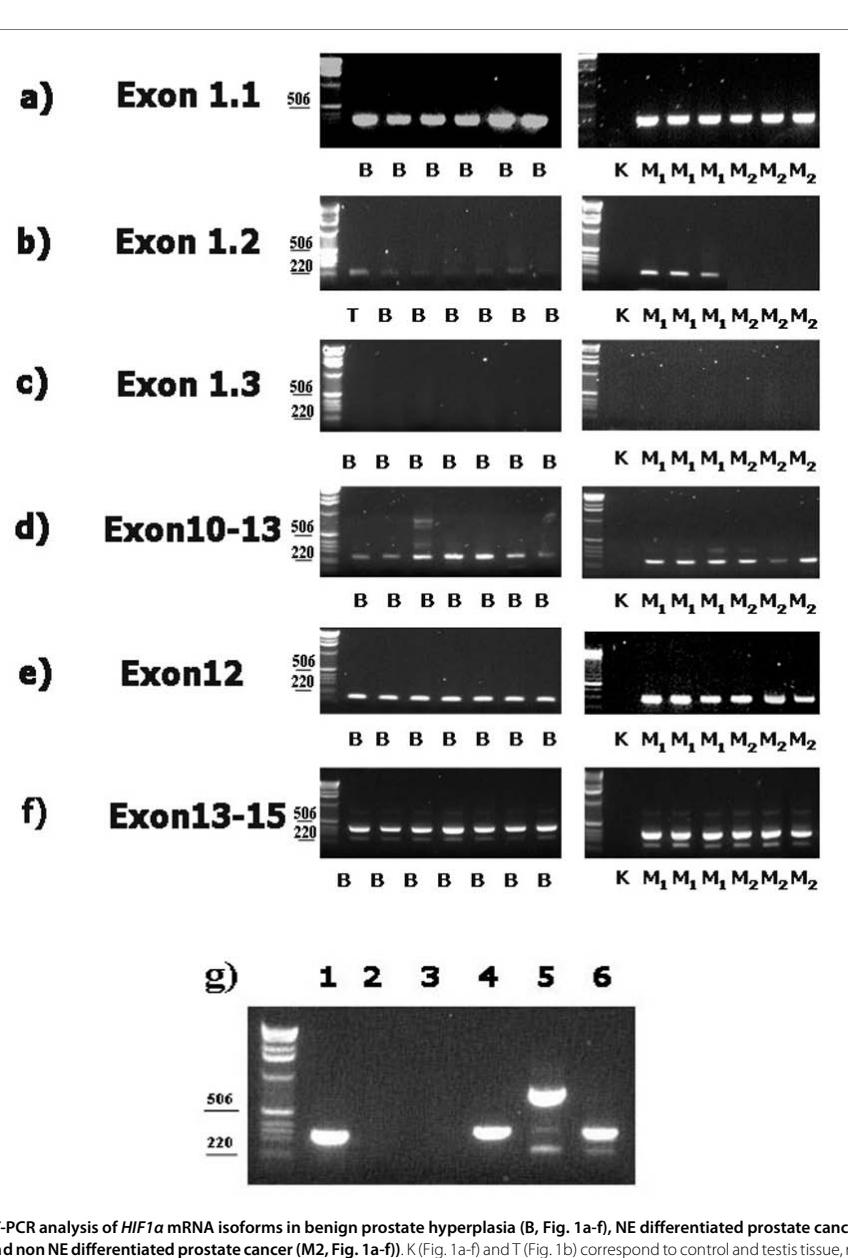
**RT-PCR analysis of *HIF1α *mRNA isoforms in benign prostate hyperplasia (B, Fig. 1a-f), NE differentiated prostate cancer (M1, Fig.1a-f)) and non NE differentiated prostate cancer (M2, Fig. 1a-f))**. K (Fig. 1a-f) and T (Fig. 1b) correspond to control and testis tissue, respectively. *HIF1α *isoforms in LNCaP cells (Fig. 1g), Lane 1 represents *HIF1α1.1*, lane 2 represents *HIF1α1.2*, and lane 3 represents *HIF1α1.3*. Lane 4: bands detected with primers sets located in exons 10 and 13, lane 5 includes bands from primers located in exon 12 and lane 6 corresponds to bands detected with primers located in exons 13 and 15

*HIF1α1.1 *isoform was present in both benign and malignant prostate tissues (Table 4, Fig.1a). *HIF1α1.2 *was detected in 25 out of 43 cases of prostate cancer and in all cases of benign prostate hyperplasia (Table 4, Fig.1b). This isoform was expressed variably, although the signals from benign prostate tissues were very weak (Fig. 1b). We also found a correlation between this isoform and NE differentiation in prostate tumors. Eighty sex percent of prostate tumors with NE (compared to 28% of prostate tumors without NE) expressed this isoform (Table 4). Comparing the presence of this isoform with HIF1α immunostaining showed that 92% of cases with this isoform also showed HIF1α immunostaining (Table 4). Therefore, we suspect that the HIF1α1.2 isoform represents the cytoplasmic HIF1α in NE cells. The weak signals from benign prostate tissue can be explained by the fact that NE cells are rare in benign prostate tissue, making up only about 10% of the cells in the prostate gland. In addition, we used non-NE-differentiated LNCaP cells as a control, and these cells did not express this isoform, further indicating that it represents cytoplasmic HIF1α (Fig. 1g, lane 2).

**Table 4:** The frequency of *HIF1α *isoforms in benign prostate hyperplasia and prostate cancer with and without NE.

Table 4	Total nr.	HIF1α 1.1	HIF1α 1.2	HIF1α 1.3	HIF1α 12+	HIF1α 12-	HIF1α 11-&12-	HIF1α 14+	HIF1α 14-
Benign	8	8 (100%)	8 (100%)	0	8 (100%)	8 (100%)	8 (100%)	8 (100%)	8 (100%)
Adenocarcinoma	43	43 (100%)	25 (58%)	0	43 (100%)	43 (100%)	43 (100%)	43 (100%)	43 (100%)
Adenocarcinoma immunopositive for HIF1α	26	26 (100%)	24 (92%)	0	26 (100%)	26 (100%)	26 (100%)	26 (100%)	26 (100%)
Adenocarcinoma immunonegative for HIF1α	17	17 (100%)	1 (6%)	0	17 (100%)	17 (100%)	17 (100%)	17 (100%)	17 (100%)
Adenocarcinoma with NE	22	22 (100%)	19 (86%)	0	22 (100%)	22 (100%)	22 (100%)	22 (100%)	22 (100%)
Adenocarcinoma Without NE	21	21 (100%)	6 (28%)	0	21 (100%)	21 (100%)	21 (100%)	21 (100%)	21 (100%)
LNCaP		+	-	-	+	+	+	+	+

The LNCaP cells were used as a control

*HIF1α *1.3 was not detected in any of our study cases (Table 4, Fig.1c). Since inflammation was rare in our specimen, this further verifies a report showing the presence of this isoform of HIF 1α in activated lymphocytes. However, we lacked positive control for HIF1α1.3.

Tο investigate various isoforms with alternative splicing of exons 11 to 13, we used forward primers located in the middle of exon 10 and reverse primers located in the middle of exon 13. We noted one strong band of 220 bp and a weaker band of 340 bp (Fig. 1d). Sequence analysis of the shorter band showed only part of exons 10 and 13, indicating the presence of an *HIF1α *isoform without exons 11 and 12. Sequence analysis of the weaker band of 340 bp was not possible, but the size of the band corresponds to an isoform lacking exon12. These two isoforms were found in both benign and malignant cases. In one case of benign prostate hyperplasia and three cases of malignant prostate tissue, we also detected a band at 720 bp, which might correspond to *HIF1α *containing exons 11 and 12. As a control, we analyzed LNCaP cells, which showed a strong band of 720bp (Fig. 1g, lane 5). Sequence analysis verified that the band corresponds to exons 10, 11 and 12 as well as a part of exon 13.

To further investigate the presence of exon 12 in benign and malignant prostate tissues, we designed primers located in the beginning and end of exon 12 (Table 2). We identified exon 12 in all study cases (Fig. 1e). We conclude that the first experiment using primers located in exons 10 and 13 was not optimal to detect the longer *HIF1α *isoform containing exon 12. We also conclude that in most cases of benign prostate hyperplasia and prostate cancers, the *HIF1α *isoform containing exon 12 is expressed at very low levels since LNCaP cells had high signals with the same primers (Fig. 1g, lane 5).

*HIF1α *variants with and without exon 14 (*HIF1α14+ and 14-*) were detected in all cases of benign prostate hyperplasia and prostate cancer. With primers located in exons 13 and 15, we detected two bands corresponding to these two isoforms (Fig. 1f).

LNCaP cells were used as control for analysis of *HIF1α1.1, HIF1α12+/-*, and *HIF1α14+/-*. *HIF1α 1.2 *and *1.3 *were not detected in these cells (Fig. 1g, lanes 1,2,3 and 6).

### Cytoplasmic HIF1α in NE cells from prostate tumors has an different N-terminus than the wild type protein

**Figure 2:**
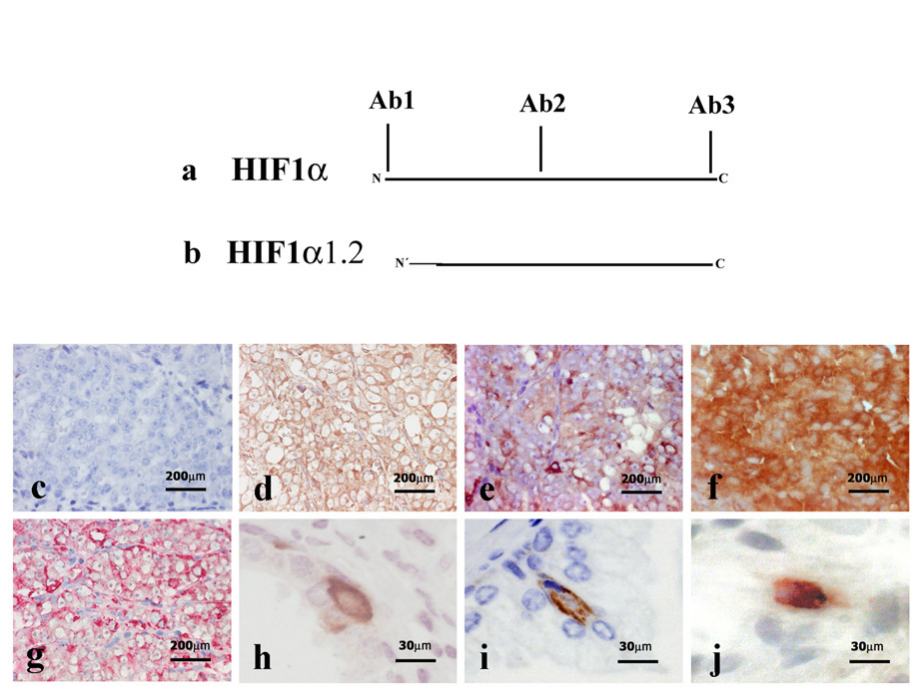
**Schematic demonstration of the three HIF1α antibodies used in this study and their epitopes (Figs. 2a and b)**. Ab1 is a polyclonal antibody raised against N-domain of wild type HIF1α and does not recognize HIF1α1.2 due to it's different N-terminal part (Fig. 2a). Ab2 and Ab3 are monoclonal and polyclonal antibodies, respectively, with epitopes in the common parts of HIF1α and HIF1α1.2 (Fig.2a and b). Immunohistochemical analysis performed on thin adjacent sections of NE-differentiated prostate cancer using the HIF1α antibodies Ab1 (Fig. 2c), Ab 2 (Fig. 2d), Ab 3 (Fig. 2e) and HIF1β antibody (Fig. 2f). Immunopositivity was detected for HIF1α with Ab2 and Ab3 while HIF1α Ab1 produced no detectable staining. HIF1β was also positive in adjacent section (Fig. 2f). Double staining of chromogranin A and androgen receptor antigens on adjacent sections (Fig. 2g) showed immunopositivity for chromogranin A (Fast red). Androgen receptor antibody (DAB, brown) produced no staining. Immunostaining of benign prostate tissue with HIF1α Ab3 showed immunopositivity in NE-like cells of benign prostate tissue (Fig. 2h). In addition, HIF1β antibody recognized benign NE-like cells in benign prostate hyperplasia (Fig. 2i). Double staining with HIF1α Ab3 and HIF1β (Fig. 2j) shows co-localization of the two proteins in NE-like cells of benign prostate hyperplasia (HIF1α Ab3 red stain, HIF1β brown stain). Panels a, b, c, d, e, f and g: 40× objective. Panels h, i and j: 60× objective.

To differentiate the isoforms of HIF1α, we used three different antibodies that recognize the N-terminus of wild type protein (Y-10 Santa Cruz, Ab1, corresponding to exons 1-2, Figs.2a and 2c), the middle section of wild type protein (NB 100-123, Ab2, corresponding to exons 10-11, Figs. 2a,b,d) and the C-terminus (Bethyl Laboratories Ab3, corresponding to exon 14, Figs. Fig. 2a,b,e). Immunostaining was performed on serial sections of benign prostate hyperplasia and HIF1α prostate cancer tissues. The antibody recognizing the N-terminus of wild type HIF1α failed to detect cytoplasmic HIF1α in malignant cells of prostate tissue (Fig.2c), whereas positive immunostaining was seen with the other two antibodies (Figs. 2d and 2e). The same results were shown with benign prostate tissue (Not shown). These results indicate that the cytoplasmic HIF1α detected in the NE cells of benign and malignant cells contains an N-terminal portion different from the wild type protein.

**Figure 3:**
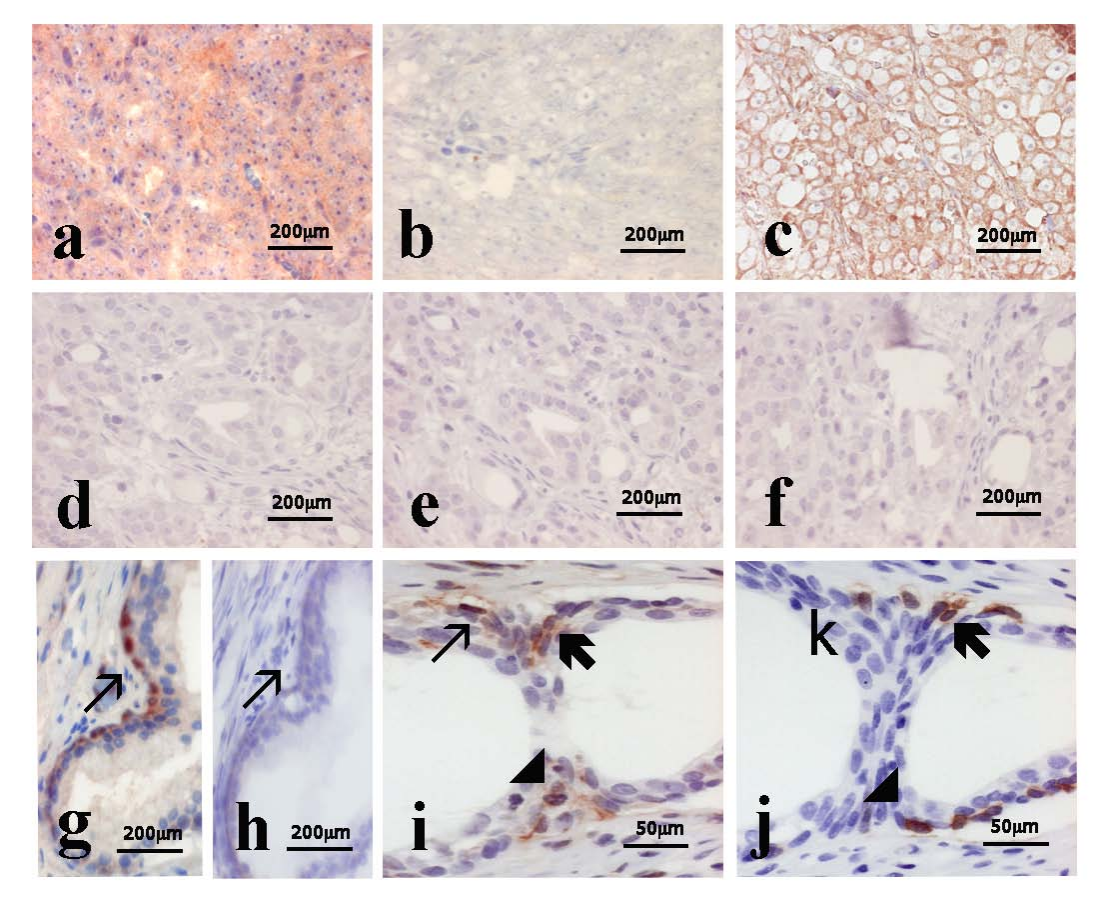
**Results of *in situ *hybridization and immunohistochemistry on thin adjacent section to detect expression of *HIF-1α1.2 *mRNA and HIF1α protein in malignant and benign prostate tissue**. *In situ *hybridization (antisense probe, Fig. 3a) and immunostaining with HIF1α Ab2 (Fig. 3c) on thin adjacent sections of NE-differentiated prostate adenocarcinoma showed co-localization of *HIF1α1.2 *transcript and HIF-1α protein. Incubation with sense probe did not generate any detectable hybridization signals (Fig. 3b). Both *In situ *hybridization (Fig. 3d antisense) and HIF-1α Ab2 immunostaining (Fig. 3f) were negative in non-NE-differentiated prostate adenocarcinoma. *In situ *hybridization with sense probe performed on non-NE-differentiated prostate cancer (Fig. 3e) was negative. *In situ *hybridizering on benign prostate tissue showed *HIF1α1.2 *transcript in NE-like cells of benign prostate tissue (Fig. 3g, ↗). The sense probe on thin adjacent section generated no signals (Fig. 3h,↗). Furthermore, co-localization of *HIF1α1.2 *transcript (Fig. 3i,↗,◢,↖) and HIF1α protein, detected with HIF1αAb3 (Fig. 3j,↗,◢,↖) was also shown in NE-like cells of benign prostate tissue. Panels a, b, c, d, e, f, g, h, i, j: 40× objective.

### Co-localization of HIF1α and HIF1β in androgen receptor-negative benign and malignant NE-like cells of the prostate

Immunostaining for HIF1β showed cytoplasmic staining in NE-like cells of benign prostate tissue (Fig. 2i). In order to investigate whether the immunoreactive HIF1β co-localizes with HIF1α in benign prostate tissue, we selected two cases of benign prostate hyperplasia for double immunostaining. We observed co-localization of cytoplasmic HIF1α with cytoplasmic HIF1β in all NE cells of the benign prostate tissue (53 cells counted) (Fig. 2j). We also investigated the localization of HIF1α and HIF1β using immunostaining of serial sections of two cases with NE and non-NE prostate tumors. HIF1β was seen in approximately 80%-100% of HIF1α-positive NE-differentiated tumor cells (Figs. 2e and 2f). In addition, double staining in malignant HIF1α-immunopositive cells showed that these cells were immunonegative for androgen receptor (Fig. 2g). In non-NE prostate tumors we found no detectable cytoplasmic HIF1β immunostaining. Only weak nuclear HIF1β staining was observed in these tumors (data not shown).

### *In situ *hybridization shows the presence of *HIF1.2α *in NE-differentiated benign and malignant prostate cells

*In situ *hybridization was performed on serially cut paraffin sections from two cases of benign and four cases of malignant prostate cancer. Two cases of adenocarcinomas were previously shown to contain cells with cytoplasmic HIF1α and NE marker, chromogranin. The other two cases were negative for cytoplasmic HIF1α and NE marker. In adjacent sections, the majority of HIF1α positive cells in both benign and malignant cells (93% versus 89%) also displayed increased *HIF1α1.2 *specific mRNA levels (Figs. 3a,c, g, i, j). Fifty cells were counted in the benign cases, and 300 cells were counted in the malignant tissue. The HIF1α-negative cells had low or no detectable mRNA levels of *HIF1α *(Figs. 3e and 3f). These findings suggest that *HIF1α1.2 *corresponds to the isoform encoding cytoplasmic HIF1α in both benign and malignant prostate tissues.

## Discussion

HIF1α, a major transcription factor essential in tumorigenesis, has been shown to be over-expressed in many types of tumors, including prostate cancer [16-18]. Neo-angiogenesis occurs in an uncoordinated fashion in malignant tumors, leading to hypoxia, inactivation of prolyl hydroxylases and inhibition of HIF1α degradation, which, in turn, activates HIF1α [19]. HIF1α protein has also been shown to be stabilized under normoxic conditions by growth factor stimulation or due to mutations in the VHL protein [20]. The accumulation of HIF1α protein has been reported to be an early event in prostate cancer and high-grade prostatic intraepithelial neoplasia (HGPIN) [21,22]. The accumulation of wild type HIF1α should lead to its nuclear transport, unless regulatory parts are defective or missing. One possible explanation for the cytoplasmic stabilization of the protein is the presence of certain HIF1α isoforms. Indeed, in this study, we have shown that a broad range of HIF1α isoforms are present in both benign prostate hyperplasia and prostate tumors. We have shown by PCR, immunohistochemistry and *in situ *hybridization that the previously reported testis-specific isoform, *HIF1α 1.2 *corresponds to cytoplasmic HIF1α in NE-differentiated benign and malignant prostate cells. HIF1α1.2, which lacks one of the NLS domains responsible for nuclear transport, was shown to accumulate in the cytoplasm and has also been shown in a previous study to have inhibitory effects on full-length HIF in human sperms [11]. The authors of that study speculated that inhibition of full-length HIF1α might be essential for the function of hypoxic sperm. A similar function for cytoplasmic HIF1α in benign and malignant prostate cells might be considered. In addition, co-localization of cytoplasmic HIF1α and HIF1β in this study indicates that this isoform might sequester HIF1β in the cytoplasm. Other reports confirm a similar function for cytoplasmic HIF1α. Stimulation of HEK293 cells with zinc induces a cytoplasmic spliced variant of HIF1α without exon 12 [23]. This 557-amino-acid isoform was shown to sequester HIF1β in the cytoplasm and therefore to inhibit wild type HIF1α. Due to a frame shift mutation, the isoform is shortened and lacks both trans activating domains and the C-terminal nuclear localizing signal domain. Another cytoplasmic spliced variant of human HIF1α (516 amino acids in length) represents a stable, short isoform without exons 11 and 12 [14]. Similarly to the variant lacking only exon 12, this isoform was identified as a dominant negative due to its association with HIF1β in the cytoplasm.

Recent studies have shown an association between hypoxia inducible factor and androgen receptor. The androgen receptor activates HIF1α, leading to upregulation of hypoxia inducible factor gene products [24,25]. In addition, hypoxia has been shown to increase androgen receptor sensitivity [26] and androgen receptor induced expression of the human PSA gene in a prostate cancer cell line [27]. Malignant NE cells which have been shown to increase in number after hormonal treatment, are usually androgen independent and only occasionally express androgen receptor and PSA [1]. As, we have shown here, the NE differentiated tumor cells display an isoform with a possible inhibitory effect on the wild type HIF1α protein, indicating that the androgen independent malignant NE cells might also be independent of HIF1α for their survival and tumorogenesis.

Malignant NE cells have been correlated with increased microvessel density in prostate tumors and display increased expression of VEGF, an important angiogenic regulatory factor. The functional implication of cytoplasmic HIF1α and its relationship to VEGF remain to be elucidated. HIF1α is a key regulatory transcription factor of VEGF, but VEGF has also been shown to be regulated by other transcriptions factors. For example, RaIA uppregulates VEGF-C in prostate cancer cells during androgen ablation [28] and STAT3 binds to the VEGF promoter and directly regulates VEGF [29]. If cytoplasmic HIF1α in NE cells exerts an inhibitory effect on full-length HIF1α, this might indicate that VEGF is up-regulated in NE cells by other transcription factors.

## Conclusion

In summary, our results suggest that cytoplasmic HIF1α in androgen independent NE cells of benign prostate tissue and non NE differentiated prostate tumors, corresponds to HIF1α1.2 isoform. Co-localization of this isoform with immunoreactive cytoplasmic HIF1β indicates that the HIF1α 1.2 isoform, which lacks one of the protein's nuclear translocation signals, may sequester HIF1β in the cytoplasm.

## Completing interests

The authors declare that they have no competing interests.

## Authors' contributions

NM and IP conceived of the study, NM carried out the design and the experiments. NM, IP, MS, PAA participated in the analysis of results. NM wrote the manuscript. IP, MS and PAA reviewed the manuscript. All authors have read and approved the final manuscript.

## Pre-publication history

The pre-publication history for this paper can be accessed here:

http://www.biomedcentral.com/1471-2407/10/385/prepub
